# Prevalence of Musculoskeletal Injuries, Pain, and Illnesses in Elite Female Basketball Players

**DOI:** 10.3390/medicina55060276

**Published:** 2019-06-14

**Authors:** Toma Garbenytė-Apolinskienė, Saulė Salatkaitė, Laimonas Šiupšinskas, Rimtautas Gudas

**Affiliations:** 1 Institute of Sports, Faculty of Nursing, Medical Academy, Lithuanian University of Health Sciences, LT-647181 Kaunas, Lithuania; saule.salatkaite@lsmuni.lt (S.S.); laimonas.siupsinskas@lsmuni.lt (L.Š.); rimtautas.gudas@kaunoklinikos.lt (R.G.); 2 Department of Orthopedics and Traumatology, Medical Academy, Lithuanian University of Health Sciences, LT-50161 Kaunas, Lithuania

**Keywords:** risk factors, trauma in athletes, epidemiology, basketball, woman

## Abstract

*Background and Objectives:* The participation of women in sports, including basketball, is becoming increasingly common, and this increased involvement raises concerns about the potential risk of sports injuries, but there is a lack of epidemiological studies about the incidence of sports injuries in women’s basketball, especially in Europe. The aim of this study was to determine the prevalence and localizations of sport related injuries and illnesses in elite female basketball players. *Materials and Methods:* This was a retrospective study. The sample consisted of 358 elite female basketball players, with a mean age of 23.4 ± 5.93 years, participated in the study. The players were interviewed using a pre-participation health questionnaire during the 2013–2016 sport seasons in the pre-season preparation phase. *Results:* 155 health problems were reported in 358 athletes during the screening period. The most frequently injured body parts were lower limbs: more than 60%, representing an injury rate of 0.14 per athletes and a 0.2 pain rate per athlete during the study period. *Conclusions:* The main findings showed the importance of monitoring lower extremity injuries and pain to pay attention to the players, which are at risk. The occurrence of injuries and pain among female basketball players was high. The lower extremities are the most frequently injured body area in the Lithuanian Women’s Basketball League.

## 1. Introduction

Basketball is a popular sport played worldwide [1]. It is a sport characterized by actions involving running, changes of direction, lateral movements, jumping, and, in particular, the impact of landing, which are conducive to the onset of injuries [2]. The participation of women in sports, including basketball, is becoming increasingly common, and this increased involvement raises concerns about the potential risk of sports injuries.

There is a lack of epidemiological studies about the incidence of sports injuries in women’s basketball in Europe [3]. Systematic monitoring of injury and illness trends over long periods of time provides epidemiological data that are indispensable to identify and, subsequently, reduce injuries and illnesses in high-risk sports and disciplines [4].

An important step for good performance is to avoid injuries, illnesses, and pain. In 2006, musculoskeletal symptoms, including pain, were the second leading reason for physician visits in the United States [5]. Pain has been highlighted by several important governmental bodies as being of particular importance to clinicians and researchers [6]. It is a significant national burden in terms of patient suffering, expenditure, and lost productivity [7]. Pain is the symptom that changes our movement patterns and increases the risk of non-contact sports injuries. Screening positive for pain may also help prescribe targeted interventions to avoid further pain and risk of injury.

Recently, some concerns have been raised about the effectiveness of the warm-up for enhancing athletic performance and preventing injuries [8,9,10]. Warm-up before physical activity is commonly accepted to be fundamental and any priming practices are usually thought to optimize performance [11]. The ideal warm-up should allow the athletes to attain an optimal muscle temperature range that limits fatigue as much as possible while maximizing performance [12,13]. So the warm-up can influence the results of sports contests and can reduce pain, fatigue, and risk of injury [14].

For the first time in Lithuania, over a 4-year period from 2013 through 2016, we collected detailed information about the injuries of high-level basketball players, and their localization and frequency. The first stage of prevention was carried out: assessment of the degree of the problem [15]. Therefore, the aim of this study was to determine the prevalence and localizations of sports-related injuries and illnesses in elite female basketball players.

## 2. Materials and Methods

A total of 389 elite female basketball players were retrospectively studied. The inclusion criterion was that all players were healthy during the pre-season and that all of them belonged to the 8 female basketball teams competing in the 1st division Lithuanian Women’s Basketball League (LWBL). All athletes were registered at the Lithuanian basketball federation.

Over the 4-year period from 2013 through 2016, we had four regular Lithuanian basketball championship seasons. At the start of the regular Lithuanian championship season, all these athletes were asked to provide individual pre-participation information (one month before the championships) using a pre-participation health questionnaire (PHQ) [16,17,18,19,20]. The PHQ was developed to collect relevant information directly from the athletes regarding their morphological status (date of birth, height, weight), training conditions (average weekly training, fatigue), and pre-competition injury and illness [16]. The subjects of the study had to indicate in the questionnaire whether they felt pre-seasonal fatigue. The answers were indicated on a black line (10 cm length). Where 1 centimeter was equal to 1 point. Thus, fatigue was rated from 0 to 10 points. Also, in the questionnaire was a question about pain, and the players had to indicate whether they felt the pain or no. The pain intensity was measured by a visual analogue scale, drawn in the questionnaire as a 10 cm line and the responder should mark the usual pain intensity and mark the area of pain in the body chart. The PHQ was easy to understand and quick to complete. The PHQ was given to athletes in paper form by their team physicians. They were asked to complete the questionnaire either alone or with help of their team physicians, if necessary, and to return it to their team physician. In four years, 31 athletes did not return the papers.

This study was approved by Kaunas Regional Biomedical Research Ethics Committee (issued on 3 June 2014, no. BE-2-27). All players were informed about the rationale of the study and provided informed consent, with guardian consent gathered when necessary (for players aged <18 years).

### Data Analysis

Statistical analysis was performed by using the the Statistical Package for the Social Sciences, version 24.0 for Windows (IBM SPSS 24.0 IBM Corp., Armonk, NY, USA). The normality assumption of data was verified with the Kolmogorov–Smirnov test. The data that were normally distributed are presented as a mean and standard deviation (SD). The data that were not distributed normally are presented as median (min-max). ANOVA was used to compare the normally distributed variable between the four groups. Adjustment for multiple comparisons was made using the Tukey HSD test. Comparing the quantitative data that were not distributed according to normal distribution, we applied nonparametric ordinal analysis. Kruskal–Wallis (one-way ANOVA on ranks) test was used to determine statistically significant differences between the four groups of an independent not normally distributed variable. To evaluate differences between categorical factors the Pearson chi-square (*χ*2) test was used. The level of significance was set at 5% for all the tests.

## 3. Results

**Figure 1:**
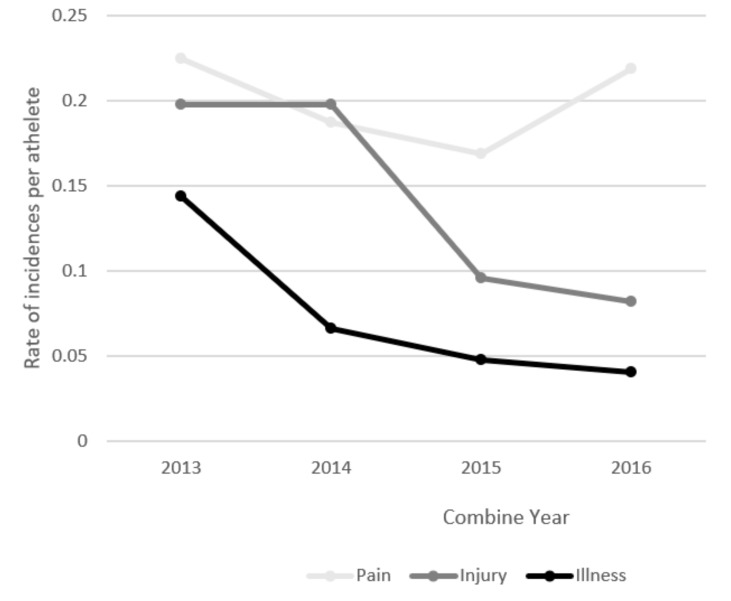
Incidence rates per athlete in the Lithuanian Women‘s Basketball League in the 2013–2016 pre-seasons (n=155 incidences of pain, injury, and illness).

In a total, 358 subjects were screened in four seasons (111 subjects in 2013; 91 subjects in 2014; 83 subjects in 2015; 73 subjects in 2016). The mean age was 23.4 ± 5.93 years, mean height was 180.1 ± 7.54 cm, mean weight was 70.85 ± 8.61 kg, mean BMI was 21.80 ± 1.89, and mean training time per week was 12.83 ± 3.52 h. Data for risk factor analysis were complete for 358 PHQs (92% of returned PHQs) (Table 1). The most common health problems in the pre-seasons are shown in Table 2. Among the 358 athletes, a total of 155 health problems were reported. In total, the lower extremities were the most frequently injured body area, accounting for 60% of all injury cases. Approximately 14% of all cases involved the head and waist, and another 8% involved the upper extremities. Incidence rates per athletes in the LWBL over 4 years are shown in Figure 1. Pain the was most common health problem during this study period before championships, except in the 2014 year, when the injury rate per athlete was higher than the pain rate. During the period of analysis, the prevalence of pain, illness, and injury was consistent (*p* > 0.05). Comparison of height, weight, body mass index, fatigue, and training time indices by the years is shown in Table 3.

## 4. Discussion

This paper analyses and discusses the risk of injuries and illnesses among athletes competing in the LWBL. It is the first step in the sequence of prevention, epidemiological, and illness surveillance studies in elite female basketball players.

Basketball requires all basic movements of high-risk sport, such as jumps, landings, acceleration, deceleration, shifts, and pivoting, which, alone, lend a potentially hurtful nature to it. During this four-year investigation, 43% of all the 358 athletes suffered from at least one injury, pain, or illness, which characterizes a high injury rate for a study. Other authors have found similar results [21,22].

Pain was a big health problem in our study (46.44%). Alemany et al. [5] described that pain was associated with slightly higher injury risk and could be a strong indicator of injury risk. So a simple indication of “pain” or ”no pain” would be a practical mean of assessing injury risk.

Concerning grouped body parts (e.g., head and waist, upper extremities, or lower extremities), a large portion of the literature addressing basketball report lower limbs as the body part with the highest incidence of injuries, specifically knee and ankle sprains [2,23]. Deitch et al. [24], in a retrospective review of 6 seasons of WNBA (Women’s National Basketball Association) and NBA (National Basketball Association) data, reported that the lower extremities (65%) were the most common site of injury, with lateral ankle sprains being the most common diagnosis. In our study, this was confirmed, where lower extremity incidences have been observed in more than 60% of athletes (injuries 30.32% and pain 29.03%). This is due to the fact that basketball is a high-intensity intermittent team sport, requiring repeated sprints, jumps, accelerations, and decelerations [25,26,27], which are conducive to the onset of injuries in these regions. Other reported common injuries in our study were head and waist areas trauma (22%), followed by upper extremity injuries (12%). In another study, approximately 15% of all game injuries involved the head and neck and another 14% involved the upper extremities [28].

Some authors claimed that athletes who presented a pre-participation health problem in the month preceding the championship were about six-times more likely to suffer a new health problem during the championship than those who did not [17]. We can state that more than one-third of players could be potentially injured. Therefore, more attention should be focused on athletes who have suffered previous injuries.

The study by Edouard et al. [17] reported that upper respiratory tract infection was the most common diagnosis (27.6% of illnesses). In our study, upper respiratory tract infection was the most common diagnosis too (19%). The difference in climatic conditions and the season (autumn) might explain these results. It is very important to pay attention to infectious disease prevention.

One study showed that training for more than 12 h per week was a risk factor for new injury [17]. In our study, the mean training time per week was not less than 12.4 h for all players. We know that fatigue can be a cause of long workouts. We are familiar that long workouts can cause a fatigue.

Practitioners are prescribing warm-ups to prevent injuries and enhance performance [29], and they claim that it could probably delay fatigue [9]. Of the 19 included articles that assessed warm-up strategies, 69% showed an improvement in sprint performance, 87.5% showed an improvement in jump performance, and 83% showed an improvement in agility performance [14]. These improvements indicated that a properly structured strategy can substantially increase athlete performance, reduce fatigue, and prevent risk of injury in many different sports including basketball.

During all pre-seasons, the players reported that they felt more than 33% fatigue. Fatigue may affect the ability of the athlete to perform over the course of a lengthy season [30]. Also, fatigue could lead to a decrease in playing performance [30].

Agel et al. [28], in their study, claimed that early season fatigue is associated with an increased risk of injury. It has been documented that fatigue reduces dynamic knee stability and, thus, increases risk of ACL injury [31]. Pre-season conditioning should be carefully planned because it can optimize performance and may reduce risk of injury.

Comparing the data by the years, we found some significant differences in training time per week and fatigue indices. Before pre-season, some of the players represent and play in national teams or other championships and had intensive training summer. These players could feel greater fatigue and their immune system could be weaker. Extended periods of intensive exercise training have been associated with progressive decreases in some immune parameters [32]. Emerging evidence indicates that inappropriate load management is a significant risk factor for acute illness and overtraining syndrome [33].

This study has some limitations. We could not exactly report that some players had risk of injury. As it was an instantaneous study, we did not analyze injuries during the seasons. Another limitation of our data collection is that all received information about health problems was self-reported. The accuracy of player data is based on reporting by athletes and team staff.

### Future Research

There is no information about the incidence and structure of sports injuries that occur to female basketball players during the season. This is the second prevention step. In this step of prevention, collection of pre-participation data on risk factors is needed. Future initiatives should include the development of preventive measures tailored to further develop scientific injury and illness surveillance systems.

## 5. Conclusions

This study highlights the importance of monitoring health problems in elite women’s basketball. The main findings showed the importance of monitoring lower extremity injuries and pain, to pay attention to the players that are at risk. We identified the lower extremities as the body part with the highest frequency of injuries and pain. The most common cause of illness were infections in the upper respiratory tract.

Practical recommendations:

- Pre-season is the perfect time to try and prepare yourself for the new season,
- The pre-participation health questionnaire can be recommended to medical staff and coaches for screening their athletes, and
- Pain screening also helps a sport medicine team pinpoint underlying musculoskeletal injuries and prescribe targeted interventions to avoid further pain and risk injury.

## Figures and Tables

**Table 1:** Pre-participation risk factor study: pre-participation health questionnaire results for 358 athletes in four seasons.

	2013	2014	2015	2016
Players, n (%)	111 (31.01)	91 (25.42)	83 (23.19)	73 (20.39)
“During the last month, have you had any difficulties participating in normal training and competition due to health problems? “				
Full participation without health problems, n (%)	63 (56.75)	56 (61.54)	62 (74.69)	50 (68.49)
Full participation, but with health problems, n (%)	31 (27.93)	26 (28.58)	16 (19.28)	17 (23.29)
Reduced participation due to health problems, n (%)	11 (9.91)	4 (4.39)	4 (4.82)	6 (8.22)
Cannot participate due to health problems, n (%)	6 (5.41)	5 (5.49)	1 (1.21)	0 (0)
For athletes who presented health problems: “To what extent has/have the health problem(s) affected your performance during training? “				
No reduction, n (%)	11 (22.90)	6 (17.14)	1 (4.76)	1 (4.35)
To a minor extent, n (%)	13 (27.45)	17 (48.57)	14 (66.67)	13 (56.51)
To a moderate extent, n (%)	10 (20.45)	6 (17.14)	3 (14.29)	7 (30.43)
To a major extent, n (%)	4 (8.75)	1 (2.86)	1 (4.76)	1 (4.35)
Cannot participate at all, n (%)	10 (20.45)	5 (14.29)	2 (9.52)	1 (4.35)

**Table 2:** Most common health problems in the Lithuanian Women‘s Basketball League, 2013–2016 pre-seasons.

Health Problem	Body Part	Number of Incidence, n	Percentage of Incidences, %
Pain	Head and waist	17	10.96
	Upper extremities	8	5.16
	Lower extremities	47	30.32
Injury	Head and waist	5	3.23
	Upper extremities	4	2.58
	Lower extremities	45	29.03
Illness	Upper respiratory tract	19	12.26
	Lower respiratory tract	2	1.29
	Cardiovascular system	1	0.65
	Blood	4	2.58
	Teeth	2	1.29
	Musculoskeletal system	1	0.65
**Total:**		**155**	**100**

**Table 3:** Comparison of height, weight, body mass index, fatigue, and training time indices by the years.

	2013	2014	2015	2016
Height, cm	180 (164–205)	180 (162–205)	180 (165–205)	180 (161–197)
Weight, kg	71.63 ± 9.29	71.94 ± 9.25	71.32 ± 8.55	69.89 ± 7.48
Body mass index, kg/m^2^	21.78 ± 1.99	22.14 ± 2.07	21.72 ± 1.81	21.70 ± 1.69
Fatigue, cm	4.28 ± 2.28	3.91 ± 2.26	3.46 ± 2.08	3.63 ± 2.06
Mean training time per week, h	12.46 ± 3.1	14.42 ± 4.04	12.46 ± 3.1	12.33 ± 3.87

Note: values are expressed as mean ± SD and median (min–max). No significantly different from each other (*p* > 0.05).
